# Semantic equivalence of the Brazilian version of the Patient Satisfaction Questionnaire (B-PSQ)

**DOI:** 10.1590/0103-6440202305074

**Published:** 2023-03-06

**Authors:** Luisa Gatti-Reis, Renata Negreiros Alvarenga, Lucas Guimarães Abreu, Saul Martins Paiva

**Affiliations:** 1 Department of Paediatric Dentistry, Universidade Federal de Minas Gerais(UFMG), Belo Horizonte, Minas Gerais, Brazil.

**Keywords:** Evidence-based dentistry, validation study, patient-centered care, patient satisfaction, malocclusion

## Abstract

The Patient Satisfaction Questionnaire (PSQ) is a self-administered instrument to assess adolescent patients' satisfaction with orthodontic treatment. A pre-existing North American instrument was further explored in the Netherlands. Semantic equivalence is part of cross-cultural adaptation and is necessary to achieve a valid and reliable instrument for a specific culture. The present study aimed to evaluate the semantic equivalence of the items, subscales, and overall PSQ between the original English version and the Brazilian Portuguese language version (B-PSQ). The PSQ has 58 items, distributed across 6 subscales: doctor-patient relationship, situational aspects of the clinic, dentofacial improvement, psychosocial improvement, dental function, and a residual category. Semantic equivalence was evaluated according to the following methods: (1) independent translations to Portuguese by two translators, both native in Brazilian Portuguese and fluent in English; (2) an expert committee drafted the first summarized version in Portuguese; (3) two independent back-translations into English by two native English-speaking translators fluent in Portuguese; (4) committee review; (5) committee drafted a summarized version of the back-translations; (6) expert committee drafted the second summarized version in Portuguese; (7) pre-test of the instrument using individual semi-structured interviews with 10 adolescents; (8) review and final version of the B-PSQ. Semantic equivalence between the original and the Brazilian versions of the questionnaire was achieved through diligent and rigorous methods, with effective translation and expert evaluations, incorporating the opinions of the target population.

## Introduction

Oral health, as defined by the FDI World Dental Federation, is “multifaceted and includes the ability to speak, smile, smell, taste, touch, chew, swallow, and convey a range of emotions through facial expressions with confidence and without pain, discomfort, and disease of the craniofacial complex” ^1^. This updated theoretical framework highlights the relevance of patient-centered care and the psychosocial function as one of the core elements of oral health, and as such, should be considered a goal of dental health care ^2^. In Orthodontics, clinician-centered outcomes have been used extensively ^3^. One study found that 63% of published orthodontic randomized clinical trials focused on occlusal changes following treatment, ignoring patient values and preferences ^3^. From a holistic perspective, a better understanding of factors related to patient satisfaction with orthodontic treatment may improve health care delivery ^4^, which confirms the necessity of evaluating this outcome in different populations.

In this context, an instrument specifically for the assessment of patient satisfaction with orthodontic treatment among adolescents has attracted the attention of scholars and clinicians ^5^. Adolescents have been the focus of recent studies assessing this outcome ^6^ ^,^ ^7^, as appliance therapy has been shown to improve their quality of life significantly ^6^. The Patient Satisfaction Questionnaire (PSQ) is a self-reported instrument, developed in the Netherlands and adapted from a pre-existing North American instrument that assessed patient satisfaction with orthognathic surgery ^5^ ^,^ ^8^. Bos et al ^5^ translated the North American instrument with 38 items from English into Dutch and added 20 questions about patient satisfaction ^5^ ^,^ ^8^. The psychometric properties have been tested in a sample of 100 individuals with a mean age of 15.81 years (SD 1.81) ^5^. Since its development, the tool has been used in Canada ^9^, and Saudi Arabia ^10^ and recently validated for use in the United Kingdom ^11^.

The use of a validated instrument is of paramount importance, as it guarantees the reliability of the results of a study ^12^. Since the PSQ was developed in another country, where different culture and language are in place, a previous validation process including its translation and cross-cultural adaptation is necessary ^13^ for use in Brazil. This process refers to the development of an instrument capable of measuring a similar phenomenon among culturally different populations, which is a requirement to obtain equivalence between the versions of the instrument in question ^13^.

To the best of our knowledge, there is no self-reported patient satisfaction instrument specific for orthodontics adapted for use in Brazil. According to the Universalist perspective, six types of equivalence must be obtained for achieving the validity of an instrument under evaluation: conceptual, item, semantic, operational, measurement, and functional equivalence ^14^. The major concern of semantic equivalence is meaning and the assessment of its translation to different languages; item equivalence refers to the underlying concept and distribution across different domains; operational equivalence refers to methods of administration of a given instrument, such as format and administration; measurement equivalence evaluates psychometric properties and functional equivalence is the sum of all previous equivalences ^15^. The present study aimed to assess the semantic equivalence of items, subscales, and overall PSQ between the English version and the Brazilian Portuguese language version.

## Material and Methods

### Study design

The present study was approved by the Institutional Review Board of the Universidade Federal of Minas Gerais (CAAE: 06898519.4.0000.5149). To participate in this study, all adolescents signed a consent form. So did the parents’/caregivers of those who were younger than 18 years. Volunteers received no compensation for their participation.

 Semantic equivalence is part of cross-cultural adaptation and is necessary to achieve a valid and reliable instrument for a specific culture ^15^. This study follows a Universalist approach, in which culture and its possible influence on the expression of concepts in different countries are taken into careful consideration ^15^. Therefore, before the evaluation of semantic equivalence, an assessment of conceptual equivalence was performed. This was necessary to evaluate whether the core concept of the instrument and its dimensions corresponded to what is expressed in Brazil, a country heavily influenced by cultural differences. This theoretical model approach was proposed by Herdman et al. ^14^ ^,^ ^15^.

A preliminary assessment of conceptual equivalence was carried out by one investigator, a native of Brazil who assessed the conceptual model of the original instrument, its domains, and relevance for the targeted population. In addition, an extensive literature review to evaluate theoretical definitions of the satisfaction construct and whether it would be relevant to complement the dimensions of the original PSQ instrument in the validation process were carried out. In the conceptual equivalence assessment, one could conclude that the domains of the original instrument corresponded in relevance to those observed in Brazilian adolescents; hence the semantic equivalence would fit an adequate instrument for future use in Brazil, warranting the necessary process for its evaluation.

**Figure 1:**
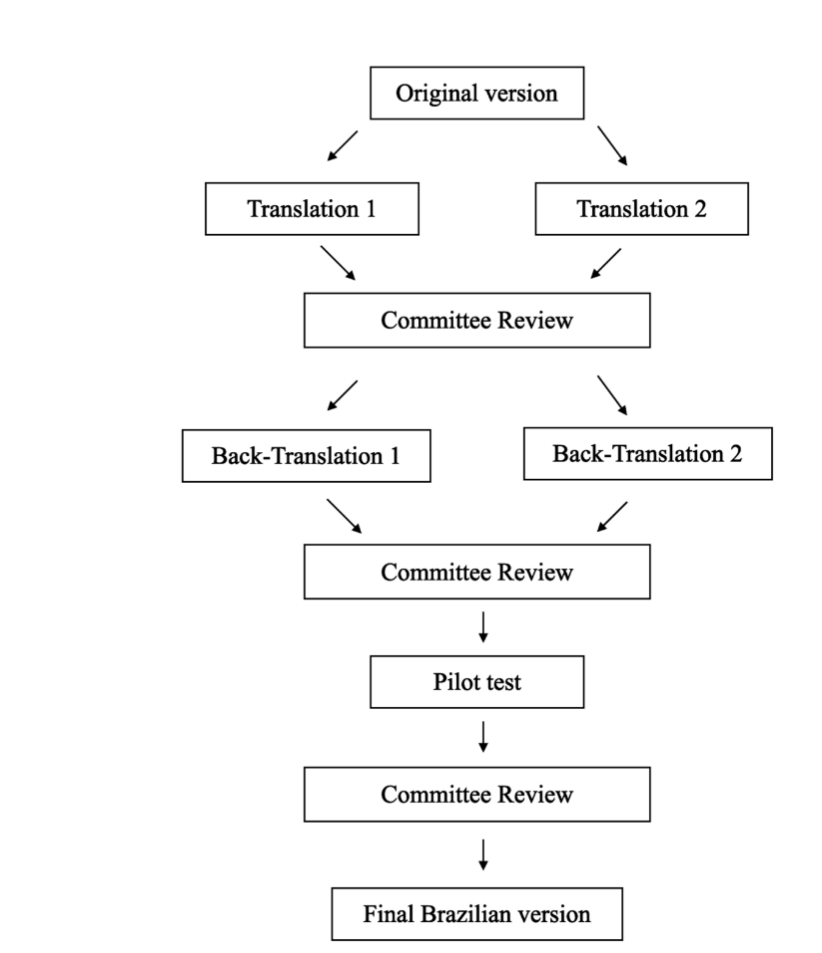
Flowchart of the semantic equivalence assessment of the Brazilian version of the Patient Satisfaction Questionnaire (B-PSQ).

To assess the semantic equivalence of the instrument, standard recommendations were followed ^13^ ^,^ ^14^. Semantic equivalence was assessed according to the sequence shown in Figure 1.

### Instrument

The PSQ is a self-reported condition-specific instrument developed to assess patient satisfaction with orthodontic treatment. It contains 58 items distributed across 6 subscales: doctor-patient relationship (11 items), situational aspects of the clinic (15 items), dentofacial improvement (9 items), psychosocial improvement (9 items), dental function (4 items), residual category (10 items). In its original version, questionnaire items were answered using a 6-point Likert scale, ranging from completely disagree (score 1) to completely agree (score 6). For items that were written in a negative sentence, the authors inverted the scoring system. In this way, patients who scored higher in the PSQ presented a higher level of satisfaction with orthodontic treatment.

### Translation

The translation of the original English version of the instrument into Brazilian Portuguese was carried out independently by two Brazilian native speakers, who were both dentists, fluent in English, and had extensive experience in epidemiological research. One of them had vast experience with the validation of health-related instruments. Before the beginning, they were informed of the nature of the instrument and also instructed to use language that could be easily understood by Brazilian adolescents of different sociodemographic backgrounds.

### Committee Review: 1^st^ Summarized version

Once the two translations had been completed, there was a subsequent meeting of an expert committee. This committee was composed of three researchers with experience in instrument development and validation, being one of them an orthodontist with vast clinical and research experience. The purpose was to review the two translated versions of the questionnaire with careful attention to its items, focused on improving comprehension by the targeted population. Shortly afterward, the committee drafted the first summarized Brazilian version of the PSQ (B-PSQ).

### Back-translation

The first summarized version of the B-PSQ was independently back-translated into English by two translators, both of whom were English native speakers and fluent in Brazilian Portuguese. Following previously established guidelines ^13^ ^,^ ^16^, the two translators were unaware of the purpose of the questionnaire and the back-translation was their only contribution to this study. This was carried out in an attempt to minimize the odds of the introduction of information bias, in addition, to allowing for the possibility of new meanings to arise, based on different points of view ^13^ ^,^ ^16^.

### Committee Review: Summarized version

When the two back-translations had been completed, the expert committee met once again to discuss the instrument. All items were reviewed, comparing the original PSQ to the two back-translated versions of the B-PSQ. A summarized version of the back-translations was drafted and compared with the original version of the PSQ, giving rise to a second summarized version in Brazilian Portuguese. This version of the B-PSQ was subsequently pilot-tested.

### Pilot test

The pilot test was carried out entirely online, in a week of November/2021 with 10 adolescents. All participants were chosen based on convenience and were from different age groups and sex, in an attempt to maintain sample representativeness. The pilot test included a survey based exclusively on the internet, followed by individual online semi-structured cognitive interviews, which were also carried out online. This summarized version in Brazilian Portuguese was tested in a convenience sample of 10 individuals representing the Brazilian population. Inclusion criteria were as follows: individuals native to Brazilian Portuguese native speakers, between 11 to 18 years, who had already completed the active phase of orthodontic treatment with fixed appliances in private orthodontic clinics. The orthodontists provided the researcher with the contact information from all the included patients, after obtaining the consent from the adolescents and their parents/guardians. Individuals with cognitive disorders reported by parents/caregivers, previous diagnosis of craniofacial anomalies, and those who had undergone orthodontic treatment associated with orthognathic surgery were excluded. Individuals with different social status were recruited. This strategy allowed one to evaluate the understanding and impact of questionnaire items across different age groups of adolescents, in addition to their different socioeconomic backgrounds. Family income was assessed according to the monthly minimum wage (MMW) nationally adopted in Brazil at the time of data collection, which corresponded approximately to US$ 220.00.

Parents/caregivers were contacted first by phone to inform about our study and ask for permission to contact their children who were invited to participate. Adolescents were contacted via text message using WhatsApp^®^ Messenger Technology, which is a platform freely available, easy to use, and widely popular in Brazil ^17^. All volunteers were given information on how to participate. Agreeing to participate, adolescents signed an online consent form, in which a detailed description of the study was provided, as follows: study goals, data collection and storage, investigator identification, and length of the survey. Then, adolescents were invited to answer an online survey, hosted on the Google Forms^®^ platform. The B-PSQ is a self-assessment questionnaire and participants were instructed to answer on their own. Subsequently, they were invited to take part in an individual interview with one of the researchers.

All semi-structured cognitive interviews were carried out online with one investigator who had been previously trained and were performed individually in Portuguese within the same week when volunteers answered the questionnaire. Each interview lasted between 15 to 30 minutes. Before the beginning, the investigator explained in detail the purpose of the investigation and the interview methods. For this online qualitative assessment, the investigator used the method known as thinking out loud ^18^ and the probe technique ^13^, discussing with adolescents the meaning of certain words that could be potentially difficult to understand. Volunteers were asked if any words would benefit from being substituted for one that could be more easily understood.

### Committee Review and final version of the B-PSQ

Following the pilot test, there was another meeting of the expert committee to discuss the test and the qualitative assessment. All suggestions given by the volunteers regarding the acceptability and understanding of questionnaire items during the interviews were carefully considered by the expert committee. Changes made to the questionnaire items were based on the perceptions of the volunteers and the consensus reached by the committee members. At last, once the committee had carefully discussed all suggestions and reached an agreement on the necessary changes, the final version of the B-PSQ was obtained (Appendix).

### Statistical analysis

Data was collected and subsequently organized in a spreadsheet - Microsoft Office Excel® for Mac (version 16.51, Redmond, WA, USA). Data analysis was carried out using the Statistical Package for Social Sciences (SPSS for Mac, version 25.0; SPSS Inc., Chicago, IL, USA), using descriptive statistics. Results were reported in absolute frequencies.

### Final Brazilian version

Following the pilot test, the perceptions and suggestions from the volunteers were discussed in a committee meeting. At last, the final version of the B-PSQ was obtained.

## Results

We conducted cognitive interviews with 10 adolescents, among whom eight were female individuals and two were male individuals. All parents who were contacted agreed with the study and agreed with the participation of their sons/daughters. The mean age of adolescents was 16.5 years (±1.7). The monthly income of the families of four adolescents was > 5 minimum wage. For six individuals, the monthly family income was ≤ 5 minimum wage. Table 1 displays the sociodemographic characteristics of the sample. After careful consideration, the expert committee agreed that all items of the original questionnaire should be maintained in the B-PSQ since they were considered relevant for the assessment of patient satisfaction with orthodontic treatment in the target population. However, the committee made a few suggestions that would make the questionnaire more adequate for use in Brazil.

**Table 1:** Sociodemographic characteristics of the sample

Parents/Caregivers	Frequency (%)
Sex	
Male	1 (10.0)
Female	9 (90.0)
Level of Schooling	
≤ 9 years	3 (30.0)
> 9 years	7 (70.0)
Family income (BMW/month)*	
> 5 minimum wages	4 (40.0)
≤ 5 minimum wages	6 (60.0)
Adolescents	
Sex	
Male	2 (20.0)
Female	8 (80.0)
Age (years)	
13	1 (10.0)
15	2 (20.0)
16	1 (10.0)
17	2 (20.0)
18	4 (40.0)

*BMW Brazilian Minimum Wage

### Portuguese Translation

There was a great similarity between the two versions translated into Brazilian Portuguese. For the words that were different, an adverb was placed instead of a noun, as in item 9, careful/carefully (careful/carefully). One difference between them that was relevant to the Portuguese language is the use of the masculine/feminine when we refer to the orthodontist who performed the treatment. The original PSQ refers to “the orthodontist”, but in Portuguese, the word itself has gender differences and implies if the practitioner is a woman or a man. To make the understanding of this word easier and avoid bias in data collection, the committee opted for masculine/feminine words and pronouns.

### Back-Translation

The two back-translations were very much similar. Occasionally, for the same word, two different synonyms were used; however, in a broader context, the meaning of the words was equal (e.g., performing my treatment/carrying out my treatment). In addition, similar to what was observed in the two Portuguese translations, one version of the back-translation was more gender-inclusive (e.g., the orthodontist always checks his/her work carefully/the orthodontist always checked his work carefully). In one item, one of the back-translations omitted an adverb (e.g., treatment took a long time/ treatment took a very long time). We opted to keep the adverbs to maintain the same idea as the original items.

## Pilot-test

Information and opinion provided by volunteers in the interviews regarding the acceptability of the questionnaire items and how easy the understanding of the item was were carefully analyzed.

In its original form, each item of the PSQ was designed to be answered in a 6-point Likert scale, ranging from completely disagree to completely agree ^5^. The expert committee achieved a consensus on the adoption of a 5-point Likert scale, using the same original endpoints, but with one mid-point “neither agree, nor disagree”. In the pilot test, the 5-point scale was used, and overall, adolescents reported that the mid-point option in the scale was helpful, mainly for items they were uncertain about. Based on their feedback, we chose to maintain a 5-point scale in the final version of the B-PSQ.

In the final version of the questionnaire, in all items that directly referred to the practitioner, the noun orthodontist was written in the singular form and the pronouns his/her were used to avoid possible confusion and misunderstanding. The expert committee also reached an agreement on other items (Box 1). The decisions are further described below:

**Box 1:**
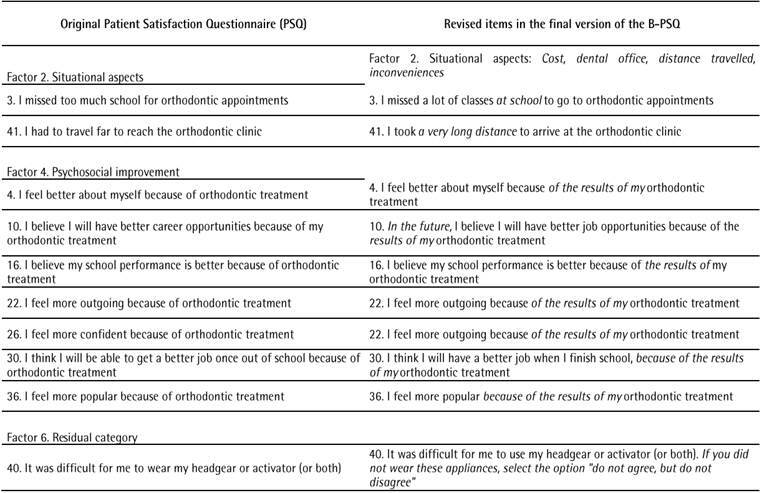
Revised items of the Brazilian Version of the Patient Satisfaction Questionnaire (B-PSQ)

### Subscale 2: Situational aspects

- Amendment to the subscale title. Originally, the title of factor 2 was “situational aspects”, but the meaning was unclear when the translation into Portuguese was performed. Thus, the following title was suggested: “Situational aspects: costs, dental office, distance traveled, inconveniences”.
- Item 3. One adolescent reported that the meaning of the phrase “I missed a lot of classes to go to orthodontic appointments” was non-specific and the researchers should be clear in stating what type of classes they were referring to. The original item in PSQ reads: “I missed too much school for orthodontic appointments”, but when translated into Portuguese, the expert committee judged “classes” as specific enough information regarding its meaning. For the final version, “classes at school” remained.
- Item 41. For two adolescents, the meaning of this item was unclear. One of them suggested the inclusion of “a very long distance”. The final version was revised to include this suggestion.

### Subscale 4: Psychosocial improvement

- Items 4, 10, 16, 22, 26, 30, 36. All items that directly associated orthodontic treatment with psychosocial improvement were amended to add the statement “because of the results”, as in “because of the results of my orthodontic treatment”. In the interviews, two adolescents expressed their concerns about how clear the meanings of these items were (e.g., how could orthodontic treatment make me feel more popular?). Moreover, adolescents reported that it could be considered incoherent, as orthodontic treatment might not be easily accessible to everybody. Based on this, the expert committee decided to make the questions more specific.
- Item 10. This item is about career opportunities. The committee agreed that, since this questionnaire is specific for adolescents, this might not be a concern for all age groups. The expression “In the future” was added to the first summarized version tested and maintained in the final version of the B-PSQ.

### Subscale 6: Residual category

- Item 40. This item refers to two treatment modalities: treatment with a headgear and treatment with an activator. In the pilot test, only one adolescent was familiar with them since he had previously worn a headgear. Because of this, the members of the committee agreed that, although this item would be maintained in the B-PSQ, a further explanation should be provided. If the adolescent had never worn such appliances, he/she would have the opportunity of choosing the mid-point option of the Likert scale.

## Discussion

To validate a pre-existing questionnaire, equivalence between the original and the target instrument must be reached ^14^ ^,^ ^15^. This study aimed to assess the semantic equivalence of the items, subscales, and overall PSQ in its original English version and the Brazilian Portuguese version. Before the adaptation process began, it must be highlighted that the original instrument was carefully assessed with respect to its conceptual equivalence to the Brazilian culture, allowing one to evaluate in what the items and the dimensions of the questionnaire corresponded to the values and beliefs of Brazilian adolescents undergoing orthodontic treatment.

The pilot test was a crucial stage in the process, as it allowed for a thorough assessment of how the culture of the target population could influence the cross-cultural adaptation of the original instrument. During development of the original instrument, a printed questionnaire was mailed to participants, who answered the questionnaire ^5^. In the present study, the same strategy would have been adopted. However, taking the epidemiological situation of Brazil in 2021, a country vastly impacted by the COVID-19 pandemic ^19^, the authors chose to carry out the pilot-test entirely online, using two easily accessible and free platforms (Google Forms^®^ and WhatsApp^®^ Messenger Technology). In this way, there was less chance of exposure and possible infection with SARS-CoV-2 in dental offices, a safer option for adolescents, dentists and researchers. When the world was hit by the pandemic, WhatsApp^®^ demonstrated the potential to become a valuable resource for telediagnosis, patient screening, and provision of recommendations for urgent care ^17^. Although this approach was an adaptation in the face of the challenges in place at the time of data collection, semantic equivalence between the original and the Brazilian version of the PSQ was successfully obtained.

One interesting decision of the expert committee was the adoption of a 5-point Likert scale with a mid-point, instead of the 6-point Likert scale of the original instrument ^5^. The use of a mid-point response on a rating scale has been the subject of discussion, seen both as a way for respondents to avoid social undesirability by not disclosing their true opinions; while also possibly leading them to choose an option that may not correspond to the truth ^20^. We highlight that for some items of the instrument, such as item 1 which mentions “Orthodontic treatment was good value for money”, not all adolescents would have a definitive opinion to answer yes or no. In items like this, the absence of a mid-point might introduce bias in data collection ^21^. This type of bias has been previously referred to as “forced choice bias”, in reference to a faulty scale that may lead respondents to choose an answer, which may not be truthful ^21^. Indeed, in the pilot study adolescents indicated that the mid-point “neither agree, nor disagree” was helpful to facilitate the reasoning that guided their answer. In addition, the PSQ with a 5-point answer scale with a mid-point had already been used elsewhere ^22^. The authors of a Dutch study ^22^ rescored the answers of the original study ^5^, which used a 6-point scale, to compare with the results of their study, obtained using a 5-point scale with a mid-point ^22^. Future studies assessing the psychometric properties of the B-PSQ should follow the scoring system proposed by Bos and Keles ^22^ to compare results across studies.

Although all items of the original questionnaire were maintained in the B-PSQ, some items needed minor modifications for confirmation of their semantic validity regarding item equivalence, acceptability, and relevance ^14^. In subscale 4, items associating orthodontic treatment with psychosocial improvement needed a revision to improve their acceptability. For instance, one adolescent reported that to judge someone’s appearance based on the chance of undergoing orthodontic treatment could be unfair, as fixed appliance therapy might not be easily available to everyone. One way to make the item clearer and more acceptable was to associate the psychosocial benefits with the results of the treatment instead. Item 40 needed a revision to maintain its relevance since the type of appliances (headgear/activator) would not be relevant to those who had not worn the orthodontic devices previously.

The cross-cultural adaptation of pre-existing instruments has indeed certain advantages. First is the availability of a standard measure that can be used in groups from different cultural contexts, allowing for the comparison of data among distinct populations. Moreover, the adaptation of a psychometric instrument is a less costly and less time-consuming process when compared to the development of a new instrument ^13^. Our study has some limitations that must be recognized. While operational equivalence was achieved between the PSQ and the B-PSQ, there was a change in operationalization: the original instrument was a postal questionnaire ^5^, and the B-PSQ was web-based administered. Data from web-based surveys should be interpreted with caution since the results produced by this data collection method may be impacted by selection bias, as the population who uses the internet might not represent the general population, having limited generalization power ^23^. However, a previous recommendation has indicated that studies should use web-based data collection if the topic of interest is suitable for the target population ^23^. We consider the topic of the current study, patient satisfaction, to be of interest to adolescent patients and, therefore, suitable for a web-based survey, especially in view of the popularity and widespread use of the WhatsApp application among Brazilian adolescents ^17^. Another point we should consider limitation regards the characteristics of the sample and the susceptibility of web-based surveys to self-selection bias ^23^. In addition, it must be noted that in the present study, a convenience sample was used with an unequal distribution of the sex of the adolescents, there 2 male and 8 female. Results should be interpreted with caution, as this sample might not be representative of the target population.

The semantic equivalence between the final version of the B-PSQ and the original version in English has been obtained successfully. Future studies investigating the psychometric properties of the B-PSQ in a representative sample of Brazilian adolescents should be encouraged.

## Appendix.

Translation of the *Patient Satisfaction Questionnaire* (PSQ), back translations, and final version in Brazilian Portuguese.
